# Analgesic and toxicological evaluation of cannabidiol-rich Moroccan *Cannabis sativa* L. (Khardala variety) extract: Evidence from an *in vivo* and *in silico* study

**DOI:** 10.1515/biol-2025-1141

**Published:** 2025-09-01

**Authors:** Hind Ibork, Zakaria Ait lhaj, Farhan Siddique, Sara El Idrissi, Farid Khallouki, Rafik El Mernissi, Lhoussain Hajji, Hanane Khalki, Mohammed Bourhia, Ahmad Mohammad Salamatullah, Ousman B. Mahamat, Khalid Taghzouti, Oualid Abboussi

**Affiliations:** Physiology and Physiopathology Team, Faculty of Sciences, Genomic of Human Pathologies Research Centre, Mohammed V University in Rabat, Rabat, Morocco; Laboratory of Organic Electronics, Department of Science and Technology, Linköping University, SE-60174, Norrköping, Sweden; Team of Ethnopharmacology and Pharmacognosy, Biology Department, Faculty of Sciences and Techniques Errachidia, Moulay Ismail University of Meknes, BP 509, Boutalamine, Errachidia, Morocco; Bioactives, Health and Environmental Laboratory, Epigenetics research team, Moulay Ismail University, Meknes, Morocco; Laboratory of Biotechnology & Sustainable Development of Natural Resources, Polydisciplinary Faculty of Beni-Mellal, Sultan Moulay Slimane University, Mghila, Beni Mellal, Morocco; Laboratory of Biotechnology and Natural Resources Valorization, Faculty of Sciences, Ibn Zohr University, 80060, Agadir, Morocco; Department of Food Science & Nutrition, College of Food and Agricultural Sciences, King Saud University, 11 P.O. Box 2460, Riyadh, 11451, Saudi Arabia; National Federation of Associations of Medical Practitioners in Chad (FENAPMT), Ministry of Health, N’Djamena, Republic of Chad

**Keywords:** neurotoxicity, *Cannabis sativa*, Khardala, toxicity, analgesia, LD_50_

## Abstract

The legalization of cannabis for industrial and medicinal purposes has significantly expanded worldwide. This study delves into the analgesic potential toxicity study of chloroformic extract from the Moroccan *Cannabis sativa* L. (*C. sativa*) cultivar, Khardala (KH extract). Our findings reveal that the lethal dose of KH extract is ≥5,000 mg/kg, with mice given 2,000 mg/kg exhibiting neurotoxic symptoms, including piloerection, aggressiveness, and fear, along with marked hepato-renal toxicity indicated by elevated levels of alanine aminotransferase, aspartate aminotransferase, total bilirubin, and creatinine in both male and female subjects. Importantly, no toxicity was observed at 250 mg/kg and 500 mg/kg doses. Remarkably, at a dose of 500 mg/kg, the KH extract demonstrated a potent analgesic effect superior to cannabidiol (CBD), suggesting a synergistic interaction among the extract’s bioactive compounds, such as CBD, cannabidivarin (CBDV), Delta 9 tetrahydrocannabinol (THC), cannabigerol (CBG), Delta 9 tetrahydrocannabivarin (THCV), and β-caryophyllene. *In silico* analysis supports these findings, showing the strong binding potential of THC, THCV, CBG, and CBDV to delta opioid receptors, with *G*-scores >−5.0 kcal/mol, highlighting the promising analgesic efficacy of this cannabis cultivar extract. This study underscores the therapeutic potential of the KH cultivar, positioning it as a promising candidate for pain management therapies.

## Graphical abstract

**Figure:**
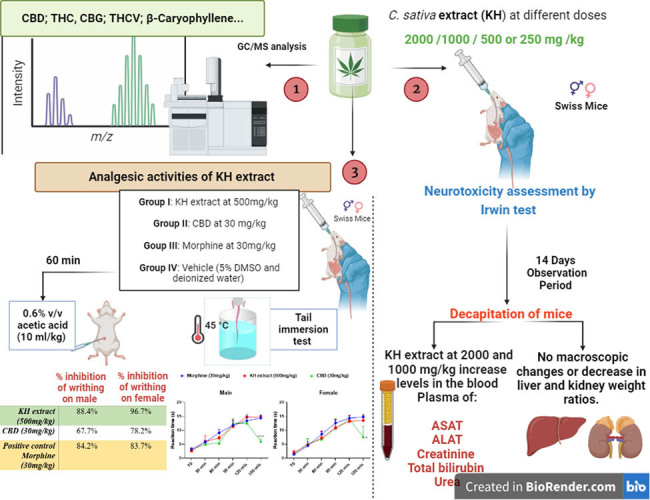

## 1 Introduction

Morocco, widely recognized as the leading global producer of *Cannabis sativa* L. (*C. sativa*), experienced a transformative shift following the legalization of cannabis for medicinal and industrial purposes in 2021, leading to a notable increase in cultivation and utilization. Among the diverse *C. sativa* cultivars, Khardala (KH) has emerged as the predominant strain, occupying over 65% of cannabis fields [1,2]. This cultivar is particularly esteemed for its high Delta 9 tetrahydrocannabinol (THC) content and its rich profile of over 400 cannabinoid compounds with profound therapeutic potential, primarily concentrated in the trichome cavity of female flowers [3].

Exploring bioactive compounds from *C. sativa*, including cannabinoids, terpenoids, flavonoids, and alkaloids, has garnered considerable interest for their pharmacological effects, particularly as analgesics [4,5]. While cannabinoids traditionally interact with the endocannabinoid system, recent studies suggest their interaction with other systems such as the opioidergic system, particularly through delta opioid receptors, elucidating potential analgesic effects [6–8]. This multifaceted interaction hints at the complex neuropharmacological mechanisms underlying cannabis’s therapeutic effects.

Among extraction methods, used to explore phytochemical plant contents, chloroform extraction has proven to be particularly effective for isolating a wide array of compounds from *C. sativa*, including cannabinoids, with minimal degradation and higher concentrations compared to alternative methods like supercritical CO_2_ or ethanol extraction, offering cost-effectiveness and accessibility for scientific investigations.

However, despite its widespread use, data on toxicity and pharmacokinetics of orally administered natural cannabinoids in humans and laboratory animals remain scarce [9]. Prior toxicity studies have predominantly focused on individual cannabinoids such as THC and cannabidiol (CBD), rather than investigating the entire spectrum of compounds present in the plant. This oversight is significant, as the synergistic interactions between the various molecules found in the cannabis plant may yield different outcomes, emphasizing the need for comprehensive evaluations of the plant’s entirety. In Morocco, the Poison and Pharmacovigilance Center reports an annual average of approximately 221 cases of drug intoxication and 430 requests for toxicological analyses, of which over 70 cases are recorded as intoxication caused by cannabinoids [10]. Hence, assessing the acute toxicity and neurotoxicity of Moroccan cannabis, particularly the KH cultivar is imperative.

CBD exhibits analgesic effects through multiple mechanisms, including modulating cannabinoid receptors: cannabinoid type-1 and type-2 receptors (CB1 and CB2), and engaging non-cannabinoid pathways [11]. CBD acts as a negative allosteric modulator of CB1 receptors, influencing receptor internalization, G protein-dependent signaling, and arrestin2 protein recruitment, particularly in regions of the central nervous system (CNS) associated with pain perception [12,13]. It also exerts an indirect effect on CB2 receptors involved in immune and inflammatory responses [14,15]. Beyond these, CBD interacts with G protein-coupled receptors like GPR3 [16], which are implicated in pain reception and neuropathic pain regulation [16]. Additionally, CBD influences serotonergic pathways via the 5-HT1A receptor [17], modulates transient receptor potential vanilloid subtype 1 (TRPV1) channels, μ and δ receptors for pain regulation, and affects the activity of enzymes like cyclooxygenases and lipoxygenases involved in inflammation [18–21]. Notably, the combination of CBD with THC is suggested to enhance CBD’s ability to interact with CB1 and CB2 receptors to elicit therapeutic responses and potentiate its analgesic properties [22]. Thus, elucidating the analgesic potential and establishing an effective dose devoid of toxicity or neurotoxicity is essential for the therapeutic optimization of specific KH extract, rich in CBD as the main non-psychoactive compound, along with THC, minor phytocannabinoids, and β-caryophyllene. Molecular docking represents a pivotal tool in drug discovery to study drug–receptor interactions and guide the development of novel therapeutics. In this study, we utilized the SWISS ADME application to investigate the potential interaction of identified cannabinoids in *C. sativa* L. KH extract against delta opioid receptors, to provide insights into the potential mechanism that may underlie their analgesic effects.

## 2 Material and methods

### 2.1 Plant material and extract preparation

The plant *C. sativa* (Khardala cultivar) was harvested from the Touanate region in Morocco (34°43′54.7″N 4°52′02.8″W; 269A) during autumn (October), and authenticated by the Scientific Institute of Rabat under the code UPOV: CANNB_SAT_SAT-68T. The fresh inflorescences were air-dried until a stable weight was achieved. A total of 2,000 g of fresh inflorescences were used, which resulted in 500 g of dried powder after air-drying, reflecting a fresh-to-dried ratio of 5:1. The dried plant material was then ground into a fine powder with a particle size of 1 µm for subsequent analysis.

The extraction of the KH extract was carried out through hot reflux extraction using a Soxhlet apparatus. To initiate the process, 20 g of the dry inflorescence powder was meticulously transferred into a cellulose extraction cartridge and inserted into the Soxhlet apparatus. Subsequently, extraction was conducted using chloroform (at a ratio of 1:10 w/v) for approximately 6 h following a defatting step with hexane. Post-extraction, the obtained extract underwent freeze-drying to eliminate any remaining solvent traces, resulting in the retention of the crude extract. The crude extract was then stored at −8°C until further analysis. The extraction yield was calculated as follows:

\[\text{Yield}( \% )\hspace{.25em}=\hspace{.25em}({M}_{0}-\text{}M)/{M}_{0}\hspace{.25em}\times \hspace{.25em}100,]\]

where *M*_0_ is the weight of the initial powder and *M* is the weight of the extract.

### 2.2 Identification of the chemical composition of KH extract by gas chromatography-mass spectrometry (GC-MS)

The chemical composition of the KH extract was determined through GC-MS analysis using a Thermo Scientific GC system (TRACE GC ULTRA), coupled with a mass spectrometer (model ISQ) and a TriPlus RSH auto-sampler. Separation of extract metabolites was performed using a TG-5MS capillary column (30 m × 0.25 mm × 0.25 μm). The injector, interface line, and detector temperatures were all set at 250°C. Helium served as the carrier gas with a flow rate of 1.5 mL/min. The acquisition mass range was configured at *m*/*z* 50–550, employing the electron-impact ionization mode (EI, 70 eV). The oven temperature was programmed to start at 80°C for 2 min, followed by a programmed rate of 8°C/min until reaching the final temperature of 280°C, which was held steady for 3 min. The extract, dissolved in chloroform, was injected into the GC-MS system without any derivatization, with a 1 μL solution injected using split mode (ratio of 10:100). Identification of the MS spectra of the separated components was conducted through comparison with the National Institute of Standards and Technology spectral library (version 2.2).

### 2.3 Animal housing and experimental design

One hundred eight male and female SWISS mice, aged 12 weeks and weighing 29 ± 0.84 g, were utilized in this study. The mice were bred in the central animal care facilities of the Faculty of Sciences at Mohammed V University in Rabat, Morocco. All animal experiments were conducted in strict accordance with the guidelines of the European Council Directive (EU2010/63), with ethical clearance number PPL/70/8647.

Before starting our experimental design, the bioactivity and drug-likeness properties of the phytocannabinoids in the *C. sativa* extract were assessed based on the published bioactivity scores and drug-likeness models of phytocannabinoids as described by Desa et al. [23]. Importantly, all compounds in the *C. sativa* extract adhered to Lipinski’s Rule of Five (molecular weight ≤500 Daltons, Log *P* ≤ 5, hydrogen bond donors ≤5, and hydrogen bond acceptors ≤10), suggesting favorable pharmacokinetic properties, including good oral bioavailability. Based on these data, we conclude that the phytocannabinoids present in our extract likely exhibit potential therapeutic properties and warrant further investigation. The only exception noted was the cannabidiol acid, which showed moderate activity in some bioactive scores.

Both sexes were included in the study as toxicity and analgesic responses can demonstrate sex-dependent variations. The 108 animals were distributed as follows.

For the toxicity assessment, 60 animals were randomly divided into one control group (*n* = 6 per sex) and four experimental groups (Group I: 2,000 mg/kg, *n* = 6 per sex; Group II: 1,000 mg/kg, *n* = 6 per sex; Group III: 500 mg/kg, *n* = 6 per sex; and Group IV: 250 mg/kg, *n* = 6 per sex).

For the analgesic potential study, 48 animals were randomly divided into one control group (*n* = 6 per sex) and three experimental groups: CBD group (*n* = 6 per sex), KH extract group (*n* = 6 per sex), and morphine group (*n* = 6 per sex).

Mice were housed in Plexiglas cages with wood shavings bedding and provided with standard diet and water *ad libitum*. Housing conditions were maintained constant, with a 12:12 light/dark cycle and a temperature of 22 ± 2°C. Each experiment involved two groups: a test group and a control group, each consisting of six mice. Female and male mice were housed separately, and female mice were non-pregnant and nulliparous. Before the experiments began, mice underwent a 1-week acclimatization period.

Animals were orally administered doses of 250, 500, 1,000, and 2,000 mg/kg of chloroform extract of KH inflorescence dissolved in a solution of deionized water and 5% dimethyl sulfoxide (DMSO). This solvent combination was chosen to prevent chemical reactions among dissolved chemicals, ensuring a purer and homogenized extract solution. The preparation of these doses followed the guidelines outlined in the OECD (2008, Document No. 425) [24,25].


**Ethical approval:** The research related to animal use has been complied with all the relevant national regulations and institutional policies for the care and use of animals, and has been approved by the Ethical Committee, of the Moroccan Association for Research and Ethics (PPL/70/8647).

### 2.4 Assessment of acute toxicity

Acute oral toxicity was evaluated following the OECD Test Guideline 425 [25], known as the Up-and-Down Procedure (UDP). The initial dose of KH extract administered was 2,000 mg/kg, as per the protocol’s recommended starting point. A control group received the vehicle (5% DMSO and deionized water). No mortality occurred at this dose, prompting the use of a step-down approach to further explore the neurotoxic and behavioral effects at lower doses. Subsequent doses of 1,000, 500, and 250 mg/kg were administered to additional groups of animals (6 per dose, equally distributed by sex) to determine the lowest dose at which no toxicity was observed. Behavioral and neurotoxic effects were recorded over a 24 h observation period using the Irwin procedure [26].

Before the gavage, the mice underwent fasting for 6 h. Their weights were then measured, and the test substance was administered. Food was withheld for 1.5 h following extract administration. The mice were monitored continuously during the initial 4 h, as well as at 24 h and 14 days post-exposure. During this observation period, changes in body weight (BW) and mortality were documented.

At the end of the 14-day observation period, the animals were fasted overnight (approximately 18 h) on the last day of the experimental protocol before blood samples were collected in clot-activator vacutainer tubes for biochemical analysis. The animals were then anesthetized and sacrificed. Subsequently, their livers and kidneys were dissected and weighed.

### 2.5 Assessment of neurotoxicity of KH extract according to Irwin procedure

The evaluation of KH extract’s impact on CNS activity and physiological function was assessed following the Irwin procedure. This standardized protocol is employed to determine the minimum lethal dose of KH extract, identify the dose range for CNS responses, and observe primary effects on behavior and physiological functions [26]. During the assessment, animals receiving various doses of KH extract, alongside a control group, were monitored for neurotoxicity-specific behaviors, including seizures and tremors. Additionally, behaviors associated with CNS stimulation, such as excitation, Straub tail response, jumping, hypersensitivity to external stimuli, stereotypical behaviors, and aggression, were observed. Conversely, behaviors indicative of CNS depression, such as sedation, rolling gait, loss of balance, impaired traction, motor incoordination, reduced sensitivity to external stimuli, decreased muscle tone, akinesia, and catalepsy, were recorded. Furthermore, the evaluation encompassed observations of autonomous functions, including breathing patterns, pupil diameter, salivation, and defecation, to comprehensively assess the neurotoxic effects induced by the KH extract.

### 2.6 Biochemical parameters analysis

Following collection, the blood samples underwent centrifugation at 3,000 rpm for 10 min at 4°C. The resulting plasma was then stored at −4°C until further analysis. A comprehensive panel of biochemical parameters was evaluated, including urea, creatinine, aspartate aminotransferase (AST), alanine aminotransferase (ALT), total bilirubin, total protein, cholesterol, uric acid, and albumin. Additionally, the ionogram profile, encompassing sodium (Na^+^), calcium (Ca^+^), chloride (Cl^−^), and potassium (K^+^), was determined. Analysis of these parameters was conducted using the Abbott Architect c8000 autoanalyzer, following the manufacturer’s instructions (Abbott, USA) [27].

### 2.7 Analgesic activities of KH extract

#### 2.7.1 Determination of the analgesic activity of KH extract

To investigate the peripheral analgesic effect of the KH extract, a dose of 500 mg/kg was selected based on the toxicity and neurotoxicity study findings. The evaluation was conducted using the acetic acid-induced writhing method described by Rashid et al. [28]. In this setup, experimental mice were randomly assigned to four groups: Group I, received vehicle (5% DMSO and deionised water); Group II, received an oral treatment of 500 mg/kg of KH extract; Group III, received an oral administration of CBD at 30 mg/kg; and Group IV, received oralmorphine at 30 mg/kg BW as positive control.

#### 2.7.2 Writhing test

Writhing was induced in mice by administering an intraperitoneal injection of 0.6% v/v acetic acid (10 mL/kg) 60 min after drug treatment. Each animal was closely monitored, and the number of writhes was recorded for 30 min, starting 5 min after writhing induction. Subsequently, the average number of writhes and the percentage inhibition of writhing were calculated, serving as indicators of analgesic activity. The calculation followed the equation outlined by Rashid et al. [28]:

\[ \% \text{writhing inhibition}\hspace{.25em}=\hspace{.25em}(({W}_{\text{c}}\hspace{.5em}\mbox{--}\hspace{.5em}W)/{W}_{\text{c}})\hspace{.25em}\times \hspace{.25em}100,]\]

where *W*_c_ is the mean number of writhes in the control group and *W* is the mean number in the experimental group (KH extract, CBD, or morphine).

#### 2.7.3 Tail immersion test

This test consists of immersing the tail of a mouse in warm water at 45°C so that the tail whips or a whole-body twitch occurs. During the experiment, the tail of the mice was placed in a water bath heated to 45 ± 0.5°C with a cut-off time of 15 s to avoid damage to the tail skin tissue [29–32]. We first measured the baseline latency (baseline latency response) for the mouse to remove the distal half of the tail after immersion in the heated water. The mice were dosed with the extract, CBD, or morphine by gavage, and the post-treatment latency responses were determined 150 min after gavage at 30 min intervals (30, 60, 90, 120, and 150 min). Furthermore, a negative control group of male and female mice was included, administered orally with vehicle (5% DMSO and deionized water), in this tail immersion test to avoid potential confounding effects and ensure that any observed variations in response could be attributed to the treatments rather than the handling or administration procedures.

### 2.8 Statistical analysis and data management

GraphPad 8.0 was used for statistical analysis. The normality of the data was evaluated using the Kolmogorov–Smirnov test. As all data were normally distributed, results were subjected to two-way ANOVA or two-way ANOVA repeated measures, followed by Bonferroni’s multiple comparisons when significance was observed. The level of significance was set at *p* < 0.05. Data from anti-analgesic activities were expressed as mean ± standard error of the mean (SEM) of biological replicates (*n* = 6 per sex), following OECD guidelines [25]. For the analgesia experiment the sample size (*n* = 6 per sex) was defined based on resources [33,34].

Acute oral toxicity data were qualitatively and quantitatively analyzed according to OECD guideline document No. 425 [25].

### 2.9 Molecular docking methodology

To investigate the analgesic activity, the X-ray crystal structure of the active delta-opioid receptor in complex with agonist molecule DPI-287 (PDB ID: 6PT3) with a resolution of 3.30 Å [35] was imported onto the workspace of Schrodinger (http://www.schrodinger.com). The protein structure was optimized at pH 7 coupled with restrained minimization using OPLS-2005 force field by employing Maestro’s protein preparation wizard. The receptor grid file, centered on the crystallized ligand, was generated with the Receptor Grid Generation module to define the receptor’s active site precisely.

The 2D structures of investigated ligands were retrieved in Structure-Data File format from the PubChem database and were prepared with the LigPrep module. The ionization states were generated at pH 7.0 ± 2.0 using Maestro’s Epick tool, employing the Hammet and Taft methodologies for accuracy. The ligands were then subjected to an energy minimization process by utilizing the OPLS-2005 forcefield to achieve the most energetically favorable geometry. The prepared ligands were docked using Glide SP scoring with an OPLS-2005 force field to find each ligand’s optimal conformational pose [36]. The *G*-score and E-model scores were calculated and then juxtaposed with the scores of crystallized ligands to evaluate the binding affinities of investigated ligands.

## 3 Results

### 3.1 Extraction yield and chemical composition of the KH extract evaluated by GC-MS

**Figure 1:**
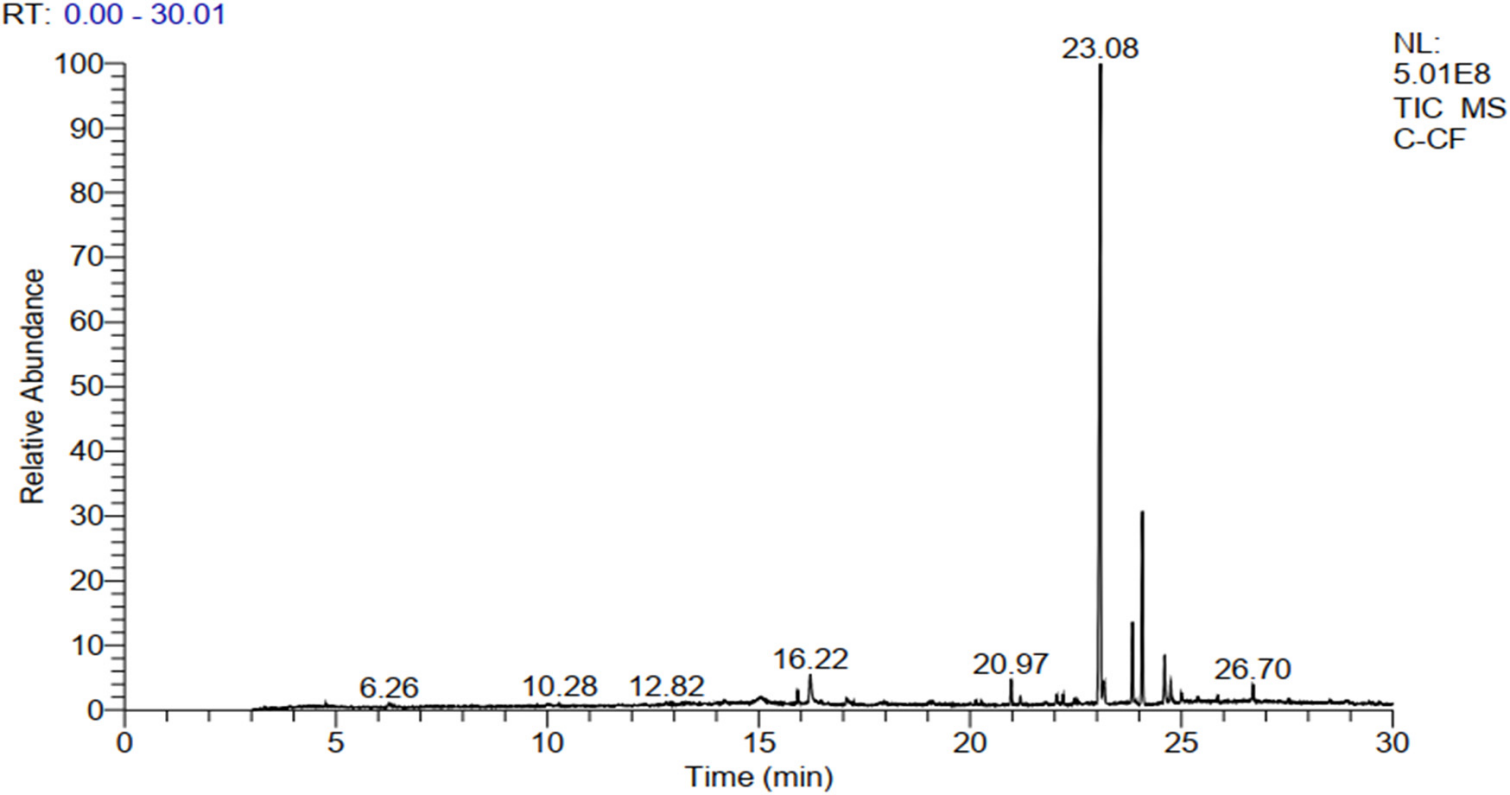
GC-MS chromatogram showing the major bioactive compounds of the KH extract.

The extract yield obtained from the hot reflux extraction by Soxhlet was calculated to be 2.45%. As illustrated in Figure 1 and Table 1, the GC-MS analysis revealed a diverse array of phytoconstituents in this extract. These include terpenoids such as neophytadiene and β-caryophyllene, alongside several cannabinoids like cannabidivarin (CBDV), THC, Delta 9 tetrahydrocannabivarin (THCV), cannabigerol (CBG), cannabinol (CBN), and CBD. Notably, CBD emerged as the predominant non-psychoactive compound, constituting approximately 60% of the peak area in the total chromatogram (with a retention time [RT] of 23.08 min).

**Table 1:** Compounds identified in the KH extract

Sr. no.	Identified compounds	RT	SI/RSI	MS (*m*/*z*)	Formula
1	Undecane	4.77	880/940	156	C_11_H_24_
2	4*H*-pyran-4-one, 2,3-dihydro-3,5-dihydroxy-6-methyl	6.26	829/846	144	C_6_H_8_O_4_
3	β-Caryophyllene	10.28	826/845	204	C_15_H_24_
4	4-(6,6-Dimethyl-2-methylenecyclohex-3-enylidene) pentan-2-ol	14.20	711/728	206	C_14_H_22_O
5	Neophytadiene	15.92	898/928	278	C_20_H_38_
6	CBDV	20.97	722/732	286	C_19_H_26_O_2_
7	THC isomer	22.01	873/874	314	C_21_H_30_O_2_
8	THCV	22.05	713/798	286	C_19_H_26_O_2_
9	Heneicosane	22.20	854/891	296	C_21_H_44_
10	CBD	23.08	901/904	314	C_21_H_30_O_2_
11	Delta-9-THC	24.08	851/852	314	C21H_30_O_2_
12	Heptacosane	24.52	868/896	380	C_27_H_56_
13	CBG	24.60	835/843	316	C_21_H_32_O_2_
14	CBN	24.75	723/751	310	C_21_H_26_O_2_

### 3.2 Acute toxicity and neurotoxicity effect of KH extract

As we reported, the acute toxicity test was conducted following the protocol described in the acute oral toxicity – UDP 425 protocol, specifically the limit test at 2,000 mg/kg. One animal was initially dosed, and upon survival, five additional animals were sequentially dosed, totaling six animals. Since all six animals survived without any fatalities, we concluded that the LD_50_ of KH extract is greater than 2,000 mg/kg. Based on the absence of mortality at this limited dose, we determined that the LD_50_ of the KH extract is ≥5,000 mg/kg.

**Figure 2:**
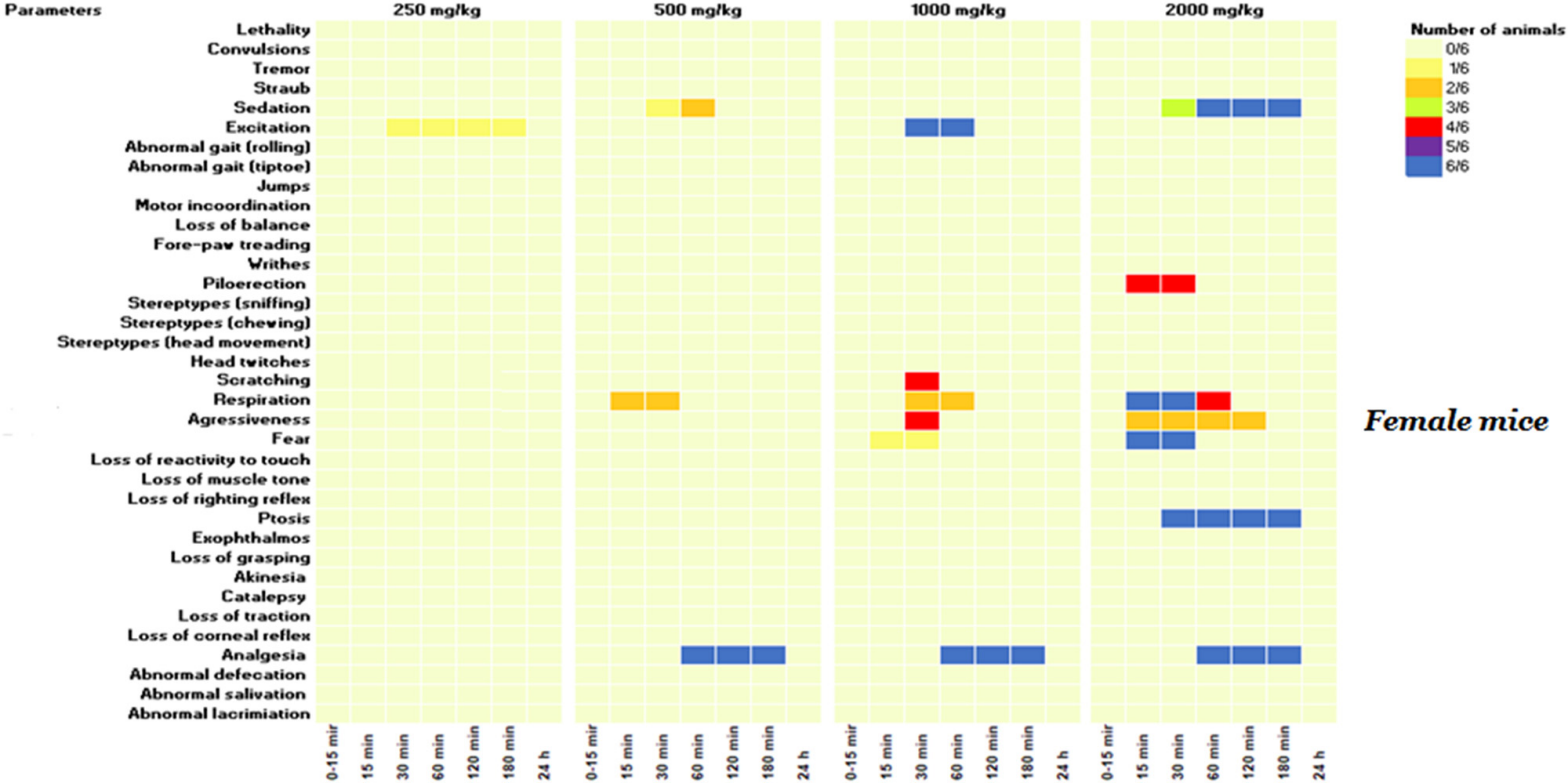
KH extract neurotoxic effects in the primary observations according to Irwin’s procedure in female mice. Data are presented as the number of animals showing neurotoxic symptoms during the test evaluated by comparison of the mean scores obtained in treated and control animals. (X/N) indicates the number of mice showing the symptoms. Observations were performed at 15, 30, 60, 120, 180 min, and 24 h after administration.

**Figure 3:**
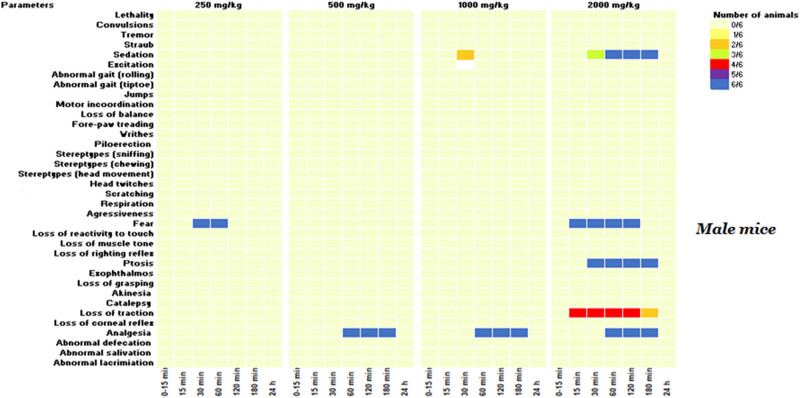
KH extract neurotoxic effects in the primary observations according to Irwin’s procedure in male mice. Data are presented as the number of animals showing neurotoxic symptoms during the test evaluated by comparison of the mean scores obtained in treated and control animals. (X/N) indicates the number of mice showing the symptoms. Observations were performed at 15, 30, 60, 120, 180 min, and 24 h after administration.

Even though no lethality was observed at 2,000 mg/kg, subsequent doses of 1,000, 500, and 250 mg/kg were administered to additional groups to determine the lowest non-toxic dose, with behavioral and neurotoxic effects recorded over 24 h. As demonstrated in Figures 2 and 3, a range of neurotoxic and behavioral changes were recorded using Irwin’s procedure. At higher doses of 2,000 and 1,000 mg/kg, the KH extract induced pronounced signs of CNS stimulation, including aggressive behavior and excitation, as well as sensorimotor deficits such as ptosis and fear. Additionally, it impacted autonomic function and peripheral activity, resulting in lacrimation, alterations in respiration rate, myosis, and mydriasis, while also demonstrating CNS depressant activity leading to sedation. Moreover, administration of KH extract at 2,000 mg/kg resulted in a motor deficit in males, characterized by a loss of traction. Based on these Irwin test results, the dose of 500 mg/kg of KH extract was selected for evaluating its potential analgesic effect.

### 3.3 Effect of KH extract on BW

**Figure 4:**
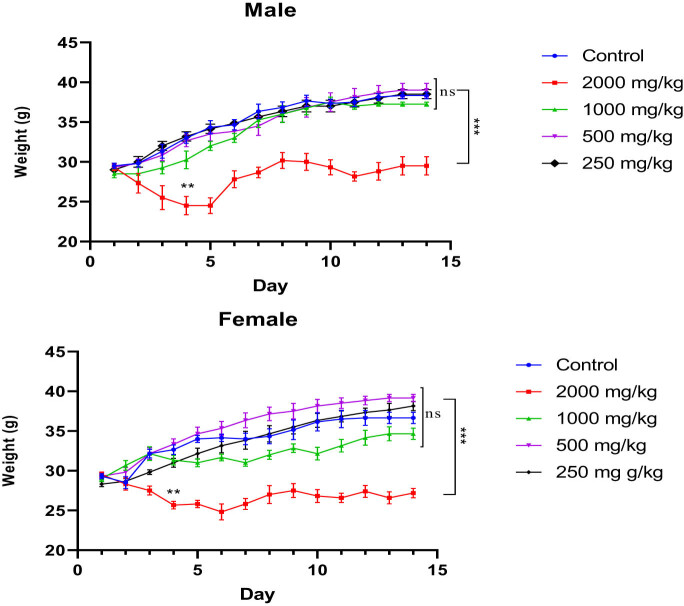
Evolution of the average BW of untreated and treated mice with the different doses (2,000, 1,000, 500, and 250 mg/kg) of KH extract of both sexes during the 14 days of experimentation. Data are expressed as mean ± SEM (*n* = 6). Data are analyzed via Bonferroni *post-hoc*; ***p < 0.001 compared to control and other dose groups.

In this study, we monitored the average BW of mice from Day 1 to Day 14. A two-way ANOVA repeated measures analysis revealed that both treatment and time significantly influenced the BW of male and female mice (*F*_(52,299)_ = 5.962, *p* < 0.0001). There were no notable changes in BW observed in untreated mice or those treated with doses of 1,000, 500, or 250 mg/kg (*p* > 0.05 vs control). However, administration of the KH extract at a dose of 2,000 mg/kg significantly decreased the BW of both male and female mice, particularly evident from the fourth and third days of the observation period (*p* = 0.002 and *p* = 0.0074 vs control, respectively) (Figure 4).

### 3.4 Effect of KH extract on liver and kidneys weight

**Figure 5:**
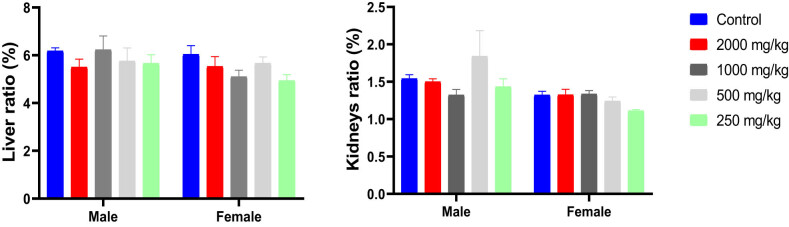
Ratio of liver and kidney weights/BWs (%) of male and female mice treated with the KH extract at 2,000, 1,000, 500, and 250 mg/kg.

Analysis of the ratio of organ weights (liver and kidney) to the BW of mice, conducted using two-way ANOVA, did not reveal any significant interaction effect between treatment and the sex of the animals (*F*_(4,47)_ = 0.8694, *p* = 0.4893 and *F*_(4,47)_ = 1.477, *p* = 0.2242 for liver and kidney, respectively). Furthermore, the results indicated no significant independent effect of treatment with KH extract at different doses on the liver and kidney ratio (*F*_(4,47)_ = 1.426, *p* = 0.2401 and *F*_(4,47)_ = 1.310, *p* = 0.2799, respectively) (Figure 5). These findings suggest that the administration of KH extract, even at its highest dose (2,000 mg/kg), does not influence the ratio of these organs to BW.

### 3.5 Effect of KH extract on biochemical parameters in mice

**Figure 6:**
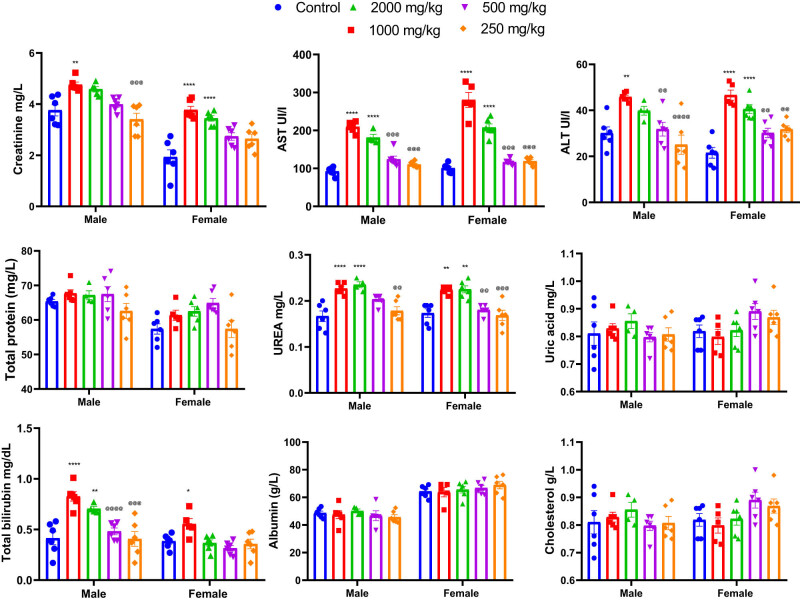
Effects of acute oral administration of KH chloroform inflorescence extract (KH extract at 2,000, 1,000, 500, and 250 mg/kg) on biochemical parameters of male and female mice (*n* = 6). Data expressed as mean ± SEM. Data are analyzed via two-way ANOVA, then Bonferroni *post-hoc*; *p < 0.05, **p < 0.01, ***p < 0.001, ****p < 0.0001 compared to control; ^@@^ *p* < 0.01, ^@@@^ *p* < 0.001, ^@@@@^ *p* < 0.0001 compared to 2,000 and 1,000 mg/kg KH treated groups. Alanine aminotransferase: ALT; aspartate aminotransferase: AST.

The results of the acute toxicity test revealed that oral administration of a single dose of 2,000 mg/kg of KH extract induced various signs of toxicity in both male and female mice. However, no deaths were recorded during the 14-day observation period. Conversely, administration of 250 and 500 mg/kg doses of KH extract to both groups of mice did not show any signs of toxicity throughout the entire 14-day observation period. To further confirm these results, the biochemical parameters of the animals’ plasma were analyzed, and the findings are depicted in Figure 6. However adverse biochemical impairments were observed in animals that received 2,000 and 1,000 mg/kg doses of KH extract. Two-way ANOVA analysis indicated a significant interaction between sex and treatment effects on the biochemical parameters, including creatinine (*F*_(4,49)_ = 2.562, *p* = 0.0499), AST (*F*_(4,47)_ = 7.230, *p* = 0.0001), ALT (*F*_(4,47)_ = 2.611, *p* = 0.0472), and total bilirubin (*F*_(4,47)_ = 4.008, *p* = 0.0070). Bonferroni multiple comparisons test indicated a significant increase in creatinine, ALT, AST, urea, and total bilirubin in male and female animals treated with 2,000 and 1,000 mg/kg KH extract compared to the untreated and treated group with 500 and 250 mg/kg of KH extract. However, no significant interactions were revealed for total protein (*F*_(4,47)_ = 0.7274, *p* = 0.5777), uric acid (*F*_(4,47)_ = 2.212, *p* = 0.0820), albumin (*F*_(4,46)_ = 1.026, *p* = 0.4038), and cholesterol (*F*_(4,47)_ = 2.212, *p* = 0.0820).

**Figure 7:**
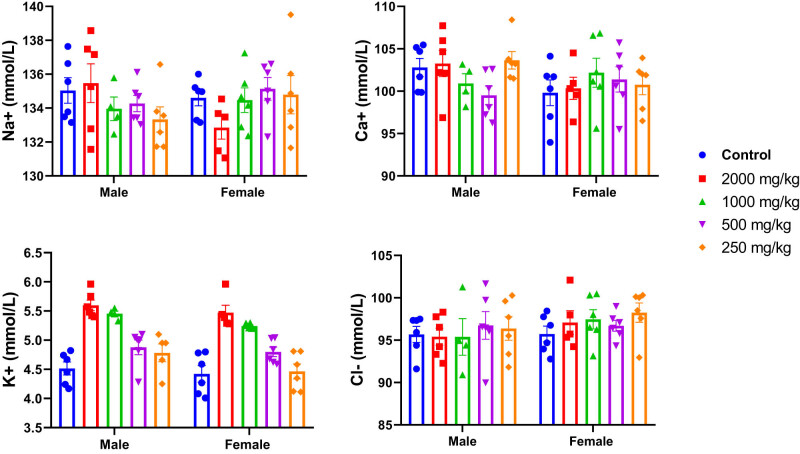
Effects of acute oral administration of KH chloroform inflorescence extract (KH extract at 2,000, 1,000, 500, and 250 mg/kg) on blood ionogram of male and female mice (*n* = 6). Data expressed as mean ± SEM.

Additionally, the potential effect of KH extract was also evaluated by assessing the ionogram profile in the blood plasma of untreated and treated mice. As illustrated in Figure 7, two-way ANOVA analysis revealed no significant interaction between sex and treatment effects on the ionogram profile of mice, indicating no effect on blood plasma levels of Na^+^ (*F*_(4,47)_ = 2.006, *p* = 0.1090), Ca^+^ (*F*_(4,47)_ = 1.661, *p* = 0.1749), K^+^ (*F*_(4,47)_ = 0.4032, *p* = 0.8054), and Cl^−^ (*F*_(4,47)_ = 0.3517, *p* = 0.8415).

### 3.6 Analgesic activities

#### 3.6.1 Effect of KH extract on writhing frequency in mice

The impact of KH extract on the acetic acid-induced writhing test was assessed using two-way ANOVA. The results revealed a significant interaction between the sex of the animals and the treatment effect (*F*_(3,40)_ = 4.291, *p* = 0.0102) (Table 2). Female and male mice administered KH extract at a dose of 500 mg/kg BW displayed a notable reduction in writhing compared to both the negative control and CBD experimental groups (*p* < 0.0001 vs both groups, Table 2). Notably, the percentage inhibition of acetic acid-induced writhing was higher in female and male mice treated with KH extract compared to the positive control group treated with morphine.

**Table 2:** Effect of the chloroform KH extract on acetic acid-induced writhing in mice; data expressed as mean ± SEM (*n* = 6, per sex)

	Negative control	KH extract (500 mg/kg)	CBD (30 mg/kg)	Positive control morphine (30 mg/kg)
Male	27.33 ± 1.05	3.16 ± 1.07***^@@^	8.83 ± 1.07***	4.33 ± 0.66***^@@^
Female	30.66 ± 1.14	1.00 ± 0.25***^@@^	6.66 ± 0.80***	5.00 ± 0.77***^@@^
% Inhibition on male	—	88.4%	67.7%	84.2%
% Inhibition on female	—	96.7%	78.2%	83.7%

***p < 0.0001 vs control group.

^@@^ *p* < 0.001 vs CBD group.

#### 3.6.2 Effect of KH extract on nociceptive-like responses of mice to hot water

**Figure 8:**
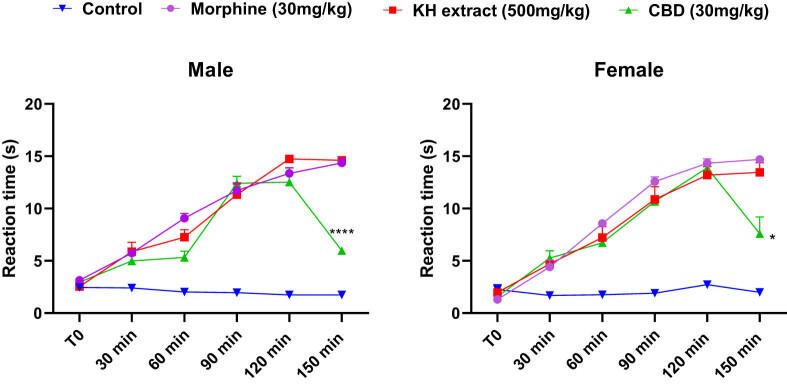
Analgesic effects of acute oral administration of KH chloroform inflorescence extract, CBD, assessed by mice reaction time in tail immersion test. Data expressed as mean ± SEM (*n* = 6). Data are analyzed via two-way ANOVA repeated measures, then Bonferroni *post-hoc*; *p < 0.05 and ****p < 0.0001 compared to morphine group and KH extract group.

The results of the tail immersion test demonstrated a time-dependent increase in nociceptive-like thresholds for both male and female mice following morphine, KH extract, or CBD treatments. Two-way ANOVA revealed significant interactions, characterized by an elongation of tail flick latency (Figure 8, *F*_(15,100)_ = 30.67, *P* < 0.0001 and *F*_(15,100)_ = 15.90, *P* < 0.0001, respectively). Notably, as demonstrated by the Bonferroni *post-hoc* multiple comparisons tests, the KH extract, CBD, and morphine-treated groups exhibited a significant antinociceptive effect during the early observation period (30 min) compared to the negative control group (*p* = 0.0025, *p* = 0.0058, and *p* < 0.0001, respectively). Moreover, both the KH and morphine groups reached their maximum effect (latency = 15 s) after 150 min, the final phase of observation. In contrast, the CBD analgesic effect notably decreased after 120 min (*p* < 0.0001 vs KH extract and morphine in male groups and *p* = 0.0147 and *p* = 0.0308 vs KH extract and morphine in female groups, respectively). These findings suggest that the KH extract possesses a potent and long-lasting antinociceptive activity compared to CBD alone.

### 3.7 Molecular docking analysis

Molecular docking analysis was performed to predict the formation of the ligand–receptor complex. The docking score was utilized to evaluate the ligand’s ability to adopt appropriate conformations within the active site of the receptor protein [37]. The analgesic activity of investigated ligands was assessed based on binding affinity with δ-opioid receptor. To function as an agonist the ligand must exhibit electrostatic or H-bonding interactions with ASP 128 deeper within the binding pocket, specifically in the extracellular region (ECL3). The interaction with ASP128 resulted in polar network rearrangements required for receptor activation and it has been observed that ligands that lack this interaction are unable to activate the receptor [35]. The Delta-9-THC demonstrated the highest binding affinity (*G*-score = −5.9 kcal/mol) and established two hydrogen bonds with the amino acid scaffold ASP 128. CBDV, THCV, and CBG also displayed H-bonding interactions with ASP128 and ILE 304 at distances ranging from 1.60 to 2.68 Å, accompanied by *G*-scores >5.0 kcal/mol. Despite the substantial binding interactions with a *G*-score of −5.2 kcal/mol, β-caryophyllene showed no interaction with ASP128, indicating that it may not activate the receptor. The detailed information on *G*-score, E-model score, H-bonding, and hydrophobic interacting residues for top hit ligands is presented in Table 3.

**Table 3:** Molecular glide score, hydrogen bonding, hydrophobic, and other interactions with distances in Angstrom for top hit ligands with δ-opioid receptor (PDB ID: 6PT3)

Ligands	Name of ligands	*G*-score (kcal/mol)	Emodel	H.B.I residue (distance, Å)	Hydrophobic and other interacting residues
03	β-Caryophyllene	−5.2	−28.71	—	ALA98, TYR129, SER131, MET132, TRP274, ILE277, CYS303, ILE304, GLY307, TYR308, SER311
06	CBDV	−5.4	−43.72	ASP128 (2.47)	ALA98, GLN105, TYR129, SER131, MET132, SER135, VAL217, TRP274, ILE277, VAL281, GLY307, TYR308, SER311
				ASP128 (2.68)	
				ILE304 (2.37)	
08	THCV	−5.7	−42.20	ASP128 (1.60)	ALA98, GLN105, TYR129, MET132, LYS214, VAL217, TRP274, ILE277, VAL281, ILE304, GLY307, TYR308, SER311
				ASP128 (2.34)	
11	Delta-9-THC	−5.9	−49.29	ASP128 (1.63)	ALA98, GLN105, TYR129, SER131, MET132, LYS214, VAL217, TRP274, ILE277, HIS278, VAL281, ILE304, GLY307, TYR308, SER311
				ASP128 (2.38)	
13	CBG	−5.1	−51.99	ASP128 (1.63)	ALA98, GLN105, TYR129, SER131, MET132, LYS214, VAL217, TRP274, ILE277, PHE280, VAL281, TRP284, LEU300, ILE304, GLY307, TYR308, SER311
				ASP128 (2.38)	
CL	Crystallized ligand	−8.2	−97.43	ASP128 (2.62)	ALA98, GLN105, MET132, LYS214, VAL217, TRP274, ILE277, HIS278, VAL281, TRP284, LEU300, ILE304, TYR308, SER311
				ASP128 (2.40)	
				ASP128 (1.94)	
				ASP128 (3.55)	
				TYR129 (2.67)	
				SER131 (2.53)	
				GLY307 (2.43)	

CL: crystallized ligand (DPI-287); H.B.I: hydrogen bonding interacting residues.

**Figure 9:**
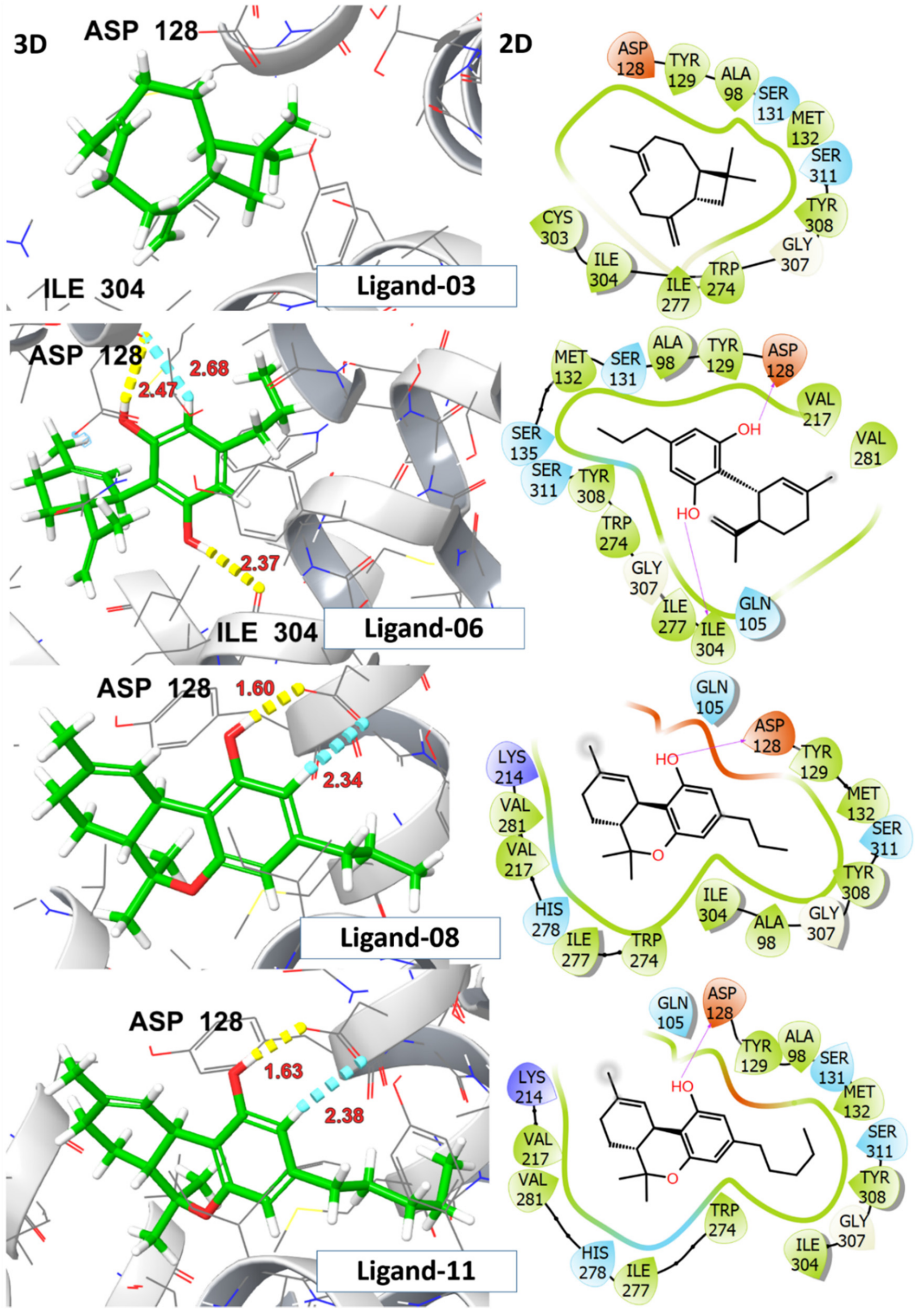
2D and 3D representation of investigated ligands 03, 06, 08, and 11 with δ-opioid receptor (PDB ID:6PT3).

**Figure 10:**
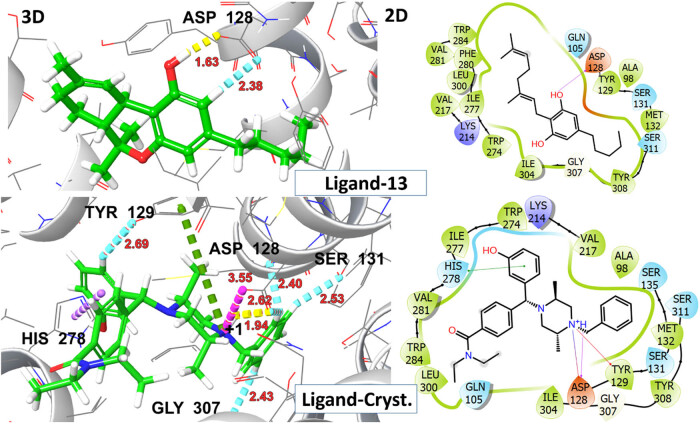
2D and 3D representation of ligands 13 and DPI-278 (co-crystallized ligand) with δ-opioid receptor (PDB ID: 6PT3).

A significant rotation of TRP284 was observed upon ligand interaction, leading to the opening of a hydrophobic pocket, comprised ILE277, HIS 278, PHE280, VAL281, TRP284, and LEU300 thereby stabilizing ligand–receptor complex through π–π stacking interactions within the binding pocket. The crystallized agonist, DPI-287, exhibited salt bridge and H-bonding interaction with ASP 128 along with other polar network aminoacid residue TYR 129 with *G*-score −8.2 kcal/mol. The aforementioned residues are involved in interactions with crystallized ligands (DPI-287), affirming a uniform binding pattern between top-hit ligands and the reference co-crystallized ligand. The visual representations of 2D and 3D interactions for ligands 03, 06, 08, and 11 are shown in Figure 9, while those for ligand 13 and the crystallized ligand DPI-287 are shown in Figure 10.

## 4 Discussion

Building upon previous studies, the chemical composition of *C. sativa* is intricate and diverse, spanning various compounds including cannabinoids, steroids, terpenoids, flavonoids, glycosides, and alkaloids [38,39]. Therefore, in this study, we specifically focused on the KH extract of *C. sativa*. Analysis conducted via GC-MS revealed the presence of diverse phytochemical metabolites. Notably, among these metabolites, we identified a diterpene neophytadiene, a sesquiterpene β-caryophyllene, and several cannabinoids such as CBDV, THC, THCV, CBG, CBN, and CBD, with CBD being the predominant non-psychoactive compound, constituting approximately 60% of the total chromatogram area.

Treatment of animals with this CBD-rich extract at various doses (2,000, 1,000, 500, and 250 mg/kg) induced differential side effects in male and female mice, which were transient and lasted less than 24 h. Notably, the high dose of 2,000 mg/kg of KH extract elicited neurotoxic side effects in males, characterized by sedation, fear, ptosis, and loss of traction. However, in addition to these effects, the extract induced piloerection and aggression in 66 and 33% of females, respectively, along with abnormal respiration rates, highlighting the greater susceptibility of female mice to the extract’s toxicity compared to males. These effects might be primarily mediated by the interaction between cannabinoids and the endocannabinoid system. THC, for example, may bind to CB1 receptors, inhibiting the release of neurotransmitters like anandamide and 2-arachidonoylglycerol (2-AG), subsequently leading to an increase in dopamine, glutamate, and acetylcholine in certain brain regions. This cascade of events influences behavioral symptoms and physiological functions such as cognition and motor movements [40,41]. Furthermore, THC induces a conformational change in the receptor, inhibiting the release of neurotransmitters like glutamate and gamma-aminobutyric acid (GABA), potentially heightening the likelihood of aggression [42]. This could explain the observed fear and aggression in mice administered with KH extract at doses of 2,000 and 1,000 mg/kg. Additionally, CBD may interact with various physiological targets, as it has been shown to inhibit CYP3A4 and CYP2D6 enzymes, resulting in reduced drug concentrations through enhanced metabolism. This interaction could amplify the effects of the molecules in the KH extract and potentially lead to adverse reactions [43,44]. Clinical observations have indeed revealed that common side effects of CBD include tiredness, diarrhea, nausea, and hepatotoxicity. It is noteworthy that these side effects are generally low in incidence and depend on the duration of exposure to CBD [45]. On another note, the neurotoxic effects induced by the KH extract (1,000 and 2,000 mg/kg) differed significantly from those reported for EU-GMP-certified *C. sativa* [46]. The certified *C. sativa*, besides its sedative impact, elicited various effects, including writhing, Straub phenomena, mild motor incoordination, stereotypes, short-duration facial tremor, and abortive seizures at doses of 300 and 2,000 mg/kg during the initial 8 h monitoring period [46]. These differences seem to be mostly mediated by the biochemical extract profile, delineating more particularly the absence of cannabinoids in their acidic forms. The authors reported that typically, cannabigerol acid (CBGA), cannabidivarinic acid, and cannabichromevarinic acid presented in the certified *C. sativa* were responsible for the Straub phenomena and writhing observed in mice [46]. Effects mediated by the pro-convulsive effect of CBGA, by acting on GABA and TRPV1 receptors [47].

Moreover, numerous studies have explored the effects of *C. sativa* extracts on neurotransmitter systems, offering a robust foundation for understanding its neurochemical impact. For example, peroral administration of ethanolic *C. sativa* extract at doses between 10 and 100 mg/kg in rats elevated glutamate and dopamine levels in the prefrontal cortex, without affecting acetylcholine levels, suggesting potential changes in emotional and cognitive performance [48]. Similarly, at a dose of 60 mg/kg, ethanolic extracts significantly increased hippocampal dopaminergic D1 receptor expression [49] Consumption of whole cannabis edibles (100 mg/kg/day every other day for 2 weeks) showed diverse neurotransmitter effects, including increased dopamine, phenylalanine, and 3,4-dihydroxyphenylacetic acid, while tryptophan decreased consistently in both brain and plasma. Additionally, 2-AG levels varied, decreasing at low doses but increasing at higher doses of complex cannabis extracts [50]. Furthermore, oral administration of chloroformic extract (50 mg/kg) in mice increased noradrenaline levels and reduced behavioral signs of stress and anxiety [51,52]. Supercritical CO_2_ extracts rich in CBD at doses of 300 mg/kg administered orally in rats modulated serotonin pathways, reducing anxiety-like behavior [53–55]. Additionally, aqueous *C. sativa* extract (200 mg/kg) administered orally in mice increased dopamine levels in the striatum, indicating potential neuroprotective properties [56–58]. These findings underscore the significant role of cannabis extracts in influencing neurotransmitter systems, with variations depending on extraction methods, doses, and routes of administration.

Moreover, to assess the extract’s toxicity, we monitored the mice’s BW daily for 14 days after the KH extract administration. Unlike the first group treated with KH extract at a dose of 2,000 mg/kg, which showed a decrease in BW from Day 4 for both males and females, the average BW of the groups treated with doses of 1,000, 500, and 250 mg/kg did not significantly differ from the control group. This effect on BW of KH extract at high doses could be attributed to the presence of THCV, as it was previously reported to decrease weight in obese mice by decreasing both body fat and leptin serum levels [59]. In addition, our findings are consistent with a recent study that reported the weight-loss effect of *C. sativa* at 10% in Sprague Dawley rats. This effect is attributed to the ability of THCV and CBD to enhance the animals’ metabolism [60].

However, during the 14 days of observation, no lethality was detected, suggesting that the LD_50_ of our extract was higher than 2,000 mg/kg. This finding is consistent with Filipiuc et al. [46] reporting that the LD_50_ value for oral administration of a certified *C. sativa* inflorescence extract (Cannabixir^®^ Medium Flos) in rats is estimated to be equal to or higher than 5,000 mg/kg. Also, Weinstein et al. [61] showed that doses of 17 and 13 mg of THC, the primary psychoactive compound, did not cause any lethality, but only affected coordination and motor skills.

The assessment of biochemical parameters, such as liver and kidney enzymes, can provide valuable insights into the potential toxicity of cannabis extract on the physiological functions of mice [62]. ALT, AST, and bilirubin levels are important indicators for assessing and monitoring healthy liver function and cytolysis [62,63].

Our results demonstrated that exposure of mice to higher cannabis extract doses (2,000 and 1,000 mg/kg) significantly increased AST, ALT, and bilirubin levels in blood plasma, similar to effects observed in rats consuming a diet with 10% *C. sativa* [60]. This hepatotoxicity may primarily be attributed to THC, as most other components found in the KH extract, such as CBD, minor cannabinoids, flavonoids, and terpenoids, are generally considered safe. In contrast, CBD, for instance, has exhibited potential hepatoprotective effects in certain animal studies. However, THC, undergoing first-pass metabolism in the liver before entering circulation [64], could lead to heightened AST and ALT levels in treated mice compared to the control group. Additionally, there was no significant increase in the biochemical parameters in the blood of mice administered with KH extract at doses of 500 and 250 mg/kg.

Additionally, this study evaluated kidney function by analyzing urea and creatinine levels. The results revealed elevated creatinine levels in the blood of mice treated with KH extract at a dose of 2,000 mg/kg compared to the control group. Also, oral administration at this dose led to a significant increase in blood urea levels, suggesting potential acute deleterious effects on the liver and kidneys [65]. Nevertheless, in line with Filipiuc et al. [66] the oral administration of KH did not result in significant alterations in total cholesterol, uric acid, albumin, and in total protein levels. This indicates that both metabolic and hepatic synthesis functions, as well as renal filtration and purification functions, remained normal, without any indications of organic damage or functional abnormalities [67].

In this respect, and based on the neurotoxicity results, we selected the 500 mg/kg BW dose to evaluate the therapeutic effects of the KH extract on mice, particularly in terms of its efficacy on pain [68]. Furthermore, according to the Irwin procedures conducted, the results demonstrated an analgesic effect in all the treated groups, suggesting the potential of KH extract as an analgesic agent [69]. To assess the analgesic effect of the KH extract, we employed two different pain assessment tests: the tail immersion test and the acid acetic-induced writhing test in mice, reflecting nociceptive and visceral pain, respectively [70–72].

Our results revealed a significant effect of KH extract in relieving acute thermal stimulus pain and reducing writhing behavior. The analgesic effect of KH extract at 500 mg/kg was comparable to that of morphine at 30 mg/kg in both male and female mice, with the effect persisting for more than 150 min (Table 2 and Figure 8). These findings are consistent with other earlier studies on the analgesic effects of cannabinoids isolated from KH extract, such as THC, CBD, CBG, and CBN [73]. Utilizing molecular docking procedures, we visualized the dynamic interactions between ligands and δ-opioid receptors, a pivotal approach in unraveling the intricate molecular mechanisms and identifying key amino acid residues (such as ASP128) responsible for δ-opioid receptor activation. Notably, ligand **11** (THC) exhibited the highest binding affinity with the target receptor, boasting a *G*-score of −5.9 kcal/mol. The cumulative analgesic effect might stem from a synergistic interaction observed among ligands 08 (THCV), 06 (CBDV), and 13 (CBG). This methodological approach not only facilitated interpreting binding preferences but also laid the groundwork for elucidating the pharmacological effects and potential analgesic applications of identified ligands targeting δ-opioid receptors. Similarly, other studies revealed that those phytocannabinoids had a higher binding affinity to opioid receptors, including µ-opioid receptor and δ-opioid receptor, with corresponding Ki (nM) values of 7,000 and 10,000, respectively, as well as with other CB receptors (CB1, CB2), [74].

The analgesic prowess of the KH extract is not solely efficient but also enduring, persisting beyond 150 min, corroborating recent research indicating that orally administered cannabinoids offer prolonged relief from allodynia in a mouse model of chronic neuropathic pain [75]. Unlike the effect of pure CBD, which decreased significantly after 150 min, this result may be attributed to the weak binding of CBD to the orthosteric site of cannabinoid receptors with a Ki in the micromolar range [76,77]. These findings were corroborated by Abraham et al. [75] demonstrating that the THC-containing extract mirrored the effect of morphine without inducing tolerance, whereas pure CBD showed a comparatively diminished effect.

This enduring effect likely owes itself to various analgesic components of KH extract, particularly CBG, previously recognized for its potent analgesic action on α-2 adrenoreceptors [78,79]. Furthermore, THCV displayed a CB2-mediated capacity to inhibit carrageenan-induced hyperalgesia and inflammation, along with both phases of formalin-induced pain behavior, achieved through the activation of CB1 and CB2 receptors in mice [80,81]. Moreover, the potential synergy between cannabinoids and terpenoids as natural remedies could bolster the KH extract’s analgesic potential. Recent studies have demonstrated that the combination of CBD and β-caryophyllene exhibits analgesic potential in alleviating chronic pain while avoiding the psychoactive effects typically associated with THC by acting on CB1 and CB2 receptors, which explains the absence of the binding of CBD and β-caryophyllene with δ-opioid receptors in our docking procedure [82–84].

Moreover, preclinical and clinical studies suggest that the combination of CBD with other compounds is more effective in reducing pain-related diseases than pure CBD alone [84,85]. In this regard, they developed medications called nabiximols such as Sativex® where the THC concentration was more than CBD (10.8–16.2 mg THC, 10.0–15 mg CBD) to help provide the best analgesic effect. However, subsequent findings indicated that using a higher co-administration dose (ranging from 29.7 to 43.2 mg THC and 27.5 to 40 mg CBD) led to side effects resembling those observed with THC alone [86,87].

Taking into account the side effects of THC, coupled with the ability of CBD to reduce them, we can conclude that the combination of THC, CBG, THCV, and β-caryophyllene with a high amount of CBD is a promising therapeutic option, which is in the line with a recent study conducted by Eeswara et al. [84]. Assuming that in this study, KH extract contains a significantly higher level of CBD than THC (60% of the total peak area of KH extract chromatogram) alongside these compounds had provided an efficient and long-lasting analgesic effect.

## 5 Conclusion

In summary, this study contributes to the growing body of research on *C. sativa*, highlighting the potential therapeutic applications of KH extract in pain management. The acute oral toxicity assessment revealed a high LD_50_ (>5,000 mg/kg) with no mortality at 2,000 mg/kg, though transient neurotoxic effects and mild hepato-renal toxicity were observed at this dose, emphasizing the need for further toxicological evaluations. At a moderate dose (500 mg/kg), KH extract exhibited significant analgesic effects in neuropathic and nociceptive pain models. This effect is likely mediated by the synergistic action of its phytoconstituents, particularly the high CBD/THC ratio with CBDV, CBG, and THCV. Molecular docking studies further supported its potential interaction with δ-opioid receptors, providing mechanistic insights into its analgesic activity.

While this study provides valuable insights into the analgesic effects of the KH extract, it is important to acknowledge certain limitations and suggest future research directions. One notable limitation is the use of mammalian models for toxicological evaluation. Although the UDP 425 protocol was rigorously followed and no animals died at the 2,000 mg/kg dose, future studies could benefit from incorporating alternative models such as *in vitro* cell cultures, computer simulations, or non-mammalian organisms (e.g., zebrafish). These alternatives could provide additional toxicological data and align with the principles of the 3Rs in animal research. Additionally, the KH extract demonstrates potential advantages over morphine concerning dependence and tolerance due to its higher CBD level and reduced THC content, along with other non-psychoactive phytocannabinoids. Hence, further research to support the current evidence base, and to feature the potential mechanisms of action related to this analgesic effect are underway. In light of this, it would be valuable to explore the crosstalk between cannabinoids and opioid receptors as we had revealed that KH extract provided the same curve slope as morphine by increasing pain thresholds in mice. Moreover, exploring the potential for reduced tolerance and dependence compared to traditional opioids could provide significant benefits for clinical pain management.

## References

[1] Afsahi K. The commodification of nature and the construction of a contested international market1. The Routledge Handbook of Post-Prohibition Cannabis Research. London, UK: Routledge; 2021. p. 241.

[2] Afsahi K. Maroc: quand la Khardala et les hybrides bouleversent le Rif. JWAPS. 2017;87:21–5.

[3] Badrana F, El Fahime EM, Mokhtari A, Soulaymani A, Safini N, Chaouni B, et al. In silico and in vitro analysis of THCA synthase gene in Moroccan Cannabis sativa. L F1000 Res. 2022;11:840.

[4] Piluzza G, Delogu G, Cabras A, Marceddu S, Bullitta S. Differentiation between fiber and drug types of hemp (Cannabis sativa L.) from a collection of wild and domesticated accessions. Genet Resour Crop Evol. 2013;60:2331–42.

[5] Bonini SA, Premoli M, Tambaro S, Kumar A, Maccarinelli G, Memo M, et al. Cannabis sativa: a comprehensive ethnopharmacological review of a medicinal plant with a long history. J Ethnopharmacol. 2018;227:300–15. 10.1016/j.jep.2018.09.004 30205181

[6] Ozdemir E. The role of the cannabinoid system in opioid analgesia and tolerance. Mini-Rev Med Chem. 2020;20:875–85. 10.2174/1389557520666200313120835 32167427

[7] Auh Q-S, Chun YH, Melemedjian OK, Zhang Y, Ro JY. Peripheral interactions between cannabinoid and opioid receptor agonists in a model of inflammatory mechanical hyperalgesia. Brain Res Bull. 2016;125:211–17. 10.1016/j.brainresbull.2016.07.009 PMC7909681 27450703

[8] Haney M. Opioid antagonism of cannabinoid effects: differences between marijuana smokers and nonmarijuana smokers. Neuropsychopharmacology. 2007;32:1391–403. 10.1038/sj.npp.1301243 17091128

[9] Poyatos L, Pérez-Acevedo AP, Papaseit E, Pérez-Mañá C, Martin S, Hladun O, et al. Oral administration of cannabis and Δ-9-tetrahydrocannabinol (THC) preparations: a systematic review. Medicina. 2020;56:309. 10.3390/medicina56060309 PMC7353904 32585912

[10] Kharchoufa L, Bouhrim M, Bencheikh N, Addi M, Hano C, Mechchate H, et al. Potential toxicity of medicinal plants inventoried in northeastern Morocco: an ethnobotanical approach. Plants. 2021;10:1108. 10.3390/plants10061108 PMC8226742 34072709

[11] Urits I, Gress K, Charipova K, Habib K, Lee D, Lee C, et al. Use of cannabidiol (CBD) for the treatment of chronic pain. Best Pract Res Clin Anaesthesiol. 2020;34:463–77. 10.1016/j.bpa.2020.06.004 33004159

[12] Laprairie R, Bagher A, Kelly M, Denovan‐Wright. Cannabidiol is a negative allosteric modulator of the cannabinoid CB1 receptor. Br J Pharmacol. 2015;172:4790–805. 10.1111/bph.13250 PMC4621983 26218440

[13] Silva-Cardoso GK, Lazarini-Lopes W, Hallak JE, Crippa JA, Zuardi AW, Garcia-Cairasco N, et al. Cannabidiol effectively reverses mechanical and thermal allodynia, hyperalgesia, and anxious behaviors in a neuropathic pain model: possible role of CB1 and TRPV1 receptors. Neuropharmacology. 2021;197:108712. 10.1016/j.neuropharm.2021.108712 34274349

[14] Narouze S. Antinociception mechanisms of action of cannabinoid-based medicine: an overview for anesthesiologists and pain physicians. Reg Anesth Pain Med. 2021;46:240–50. 10.1136/rapm-2020-102114 33239391

[15] Manzanares J, Julian M, Carrascosa A. Role of the cannabinoid system in pain control and therapeutic implications for the management of acute and chronic pain episodes. Curr Neuropharmacol. 2006;4:239–57. 10.2174/157015906778019527 PMC2430692 18615144

[16] Laun AS, Shrader SH, Brown KJ, Song Z-H. GPR3, GPR6, and GPR12 as novel molecular targets: their biological functions and interaction with cannabidiol. Acta Pharmacol Sin. 2019;40:300–8. 10.1038/s41401-018-0031-9 PMC6460361 29941868

[17] Russo EB, Burnett A, Hall B, Parker KK. Agonistic properties of cannabidiol at 5-HT1a receptors. Neurochem Res. 2005;30:1037–43. 10.1007/s11064-005-6978-1 16258853

[18] Bruni N, Della Pepa C, Oliaro-Bosso S, Pessione E, Gastaldi D, Dosio F. Cannabinoid delivery systems for pain and inflammation treatment. Molecules. 2018;23:2478. 10.3390/molecules23102478 PMC6222489 30262735

[19] Dieterle M, Zurbriggen L, Mauermann E, Mercer-Chalmers-Bender K, Frei P, Ruppen W, et al. Pain response to cannabidiol in opioid-induced hyperalgesia, acute nociceptive pain, and allodynia using a model mimicking acute pain in healthy adults in a randomized trial (CANAB II). Pain. 2022;163:1919–28. 10.1097/j.pain.0000000000002591 PMC9982727 35239547

[20] Bosquez-Berger T, Gudorf JA, Kuntz CP, Desmond JA, Schlebach JP, VanNieuwenhze MS, et al. Structure–activity relationship study of cannabidiol-based analogs as negative allosteric modulators of the μ-opioid receptor. J Med Chem. 2023;66:9466–94. 10.1021/acs.jmedchem.3c00061 PMC11299522 37437224

[21] Grogan G, Stephens K, Chou J, Timko MP, Cottler P, DeGeorge Jr BR. The mechanism of cannabichromene and cannabidiol alone versus in combination in the alleviation of arthritis-related inflammation. Ann Plast Surg. 2023;90:S408–15. 10.1097/SAP.0000000000003547 37332213

[22] Thomas A, Baillie G, Phillips A, Razdan R, Ross RA, Pertwee R. Cannabidiol displays unexpectedly high potency as an antagonist of CB1 and CB2 receptor agonists in vitro. Br J Pharmacol. 2007;150:613–23. 10.1038/sj.bjp.0707133 PMC2189767 17245363

[23] Desa S, Osman A. Hyslop RJEJoS, mathematics, technology. in silico assessment of drug-like properties of phytocannabinoids in Cannabis sativa. 2017;4:1–7.

[24] Luechtefeld T, Maertens A, Russo DP, Rovida C, Zhu H, Hartung T. Analysis of public oral toxicity data from REACH registrations 2008–2014. J Altex. 2016;33:111. 10.14573/altex.1510054 PMC5461469 26863198

[25] Development OfEC-o. Test No. 425: acute oral toxicity: up-and-down procedure. Paris, France: OECD Publishing; 2008.

[26] Roux S, Sablé E, Porsolt RD. Primary observation (Irwin) test in rodents for assessing acute toxicity of a test agent and its effects on behavior and physiological function. Curr Protoc Pharmacol. 2004;27:10. 10.1002/0471141755.ph1010s27 22294127

[27] Pauli D, Seyfarth M, Dibbelt L. The Abbott Architect c8000: analytical performance and productivity characteristics of a new analyzer applied to general chemistry testing. Clin Lab. 2005;51:31–42. 15719702

[28] Rashid M, Biswas S, Abdullah-Al-Mamun M, Huque A, Bhuiyan JR. Phytochemical screening and analgesic, anti-bacterial and cytotoxic activity evaluation of ethanol extract of Pithcellobium dulce (Roxb.) benth leaf. Asian J Pharm Clin Res. 2015;8:451–7.

[29] Luttinger D. Determination of antinociceptive efficacy of drugs in mice using different water temperatures in a tail-immersion test. J Pharmacol Methods. 1985;13:351–7. 10.1016/0160-5402(85)90017-8 3927065

[30] Sun L, Liao L, Wang B. Potential antinociceptive effects of Chinese propolis and identification on its active compounds. J Immunol Res. 2018;2018:5429543. 10.1155/2018/5429543 PMC6178491 30356413

[31] Jain M, Nema P, Jain S, Khan R, Dixena S, Jain PK, et al. To evaluate and compare the analgesic activity of piperine and extracts of Piper nigrum L. fruit. J Drug Delivery Ther. 2024;14:49–53.

[32] Luo L, Wang Y, Li B, Xu L, Kamau PM, Zheng J, et al. Molecular basis for heat desensitization of TRPV1 ion channels. Nat Commun. 2019;10:2134. 10.1038/s41467-019-09965-6 PMC6513986 31086183

[33] Charan J, Biswas T. How to calculate sample size for different study designs in medical research? Indian J Psychol Med. 2013;35:121–6. 10.4103/0253-7176.116232 PMC3775042 24049221

[34] Festing MF, Altman, Douglas G. Guidelines for the design and statistical analysis of experiments using laboratory animals. ILAR J. 2002;43:244–58. 10.1093/ilar.43.4.244 12391400

[35] Claff T, Yu J, Blais V, Patel N, Martin C, Wu L, et al. Elucidating the active δ-opioid receptor crystal structure with peptide and small-molecule agonists. Sci Adv. 2019;5:eaax9115. 10.1126/sciadv.aax9115 PMC6881160 31807708

[36] Friesner RA, Banks JL, Murphy RB, Halgren TA, Klicic JJ, Mainz DT, et al. Glide: a new approach for rapid, accurate docking and scoring. 1. Method and assessment of docking accuracy. J Med Chem. 2004;47:1739–49. 10.1021/jm0306430 15027865

[37] Meng X-Y, Zhang H-X, Mezei M, Cui M. Molecular docking: a powerful approach for structure-based drug discovery. Curr Comput drug Des. 2011;7:146–57. 10.2174/157340911795677602 PMC3151162 21534921

[38] Richter G, Hazzah T, Hartsel JA, Eades J, Hickory B, Makriyannis A. Cannabis sativa: an overview. Nutraceuticals. Academic Press; 2021 p. 603–24.

[39] Odieka AE, Obuzor GU, Oyedeji OO, Gondwe M, Hosu YS, Oyedeji AO. The medicinal natural products of Cannabis sativa Linn.: a review. Molecules. 2022;27:1689. 10.3390/molecules27051689 PMC8911748 35268790

[40] López‐Moreno JA, González‐Cuevas G, Moreno G, Navarro M. The pharmacology of the endocannabinoid system: functional and structural interactions with other neurotransmitter systems and their repercussions in behavioral addiction. Addict Biol. 2008;13:160–87. 10.1111/j.1369-1600.2008.00105.x 18422831

[41] Mechoulam R, Hanuš L. A historical overview of chemical research on cannabinoids. Chem Phys Lipids. 2000;108:1–13. 10.1016/s0009-3084(00)00184-5 11106779

[42] Copas G, Amazonas E, Brandon S. The pharmacology of cannabinoids. In Cannabis therapy in veterinary medicine: a complete guide. Cham: Springer; 2021. p. 17–59.

[43] Kowalczyk K, Lasek P, Trąbka N, Binkowska P, Demidowicz G, Lasota N, et al. Medical cannabis: mechanisms of action and therapeutic targets. J Educ Health Sport. 2024;58:176–90.

[44] Chayasirisobhon S. Mechanisms of action and pharmacokinetics of cannabis. Perm J. 2020;25:1–3. 10.7812/TPP/19.200 PMC8803256 33635755

[45] Maldonado C, Peyraube R, Fagiolino P, Oricchio F, Cuñetti L, Vázquez M. Human data on pharmacokinetic interactions of cannabinoids: a narrative review. Curr Pharm Des. 2024;30:241–54. 10.2174/0113816128288510240113170116 38288797

[46] Filipiuc L-E, Ştefănescu R, Solcan C, Ciorpac M, Szilagyi A, Cojocaru D, et al. Acute toxicity and pharmacokinetic profile of an EU-GMP-Certified Cannabis sativa L. in rodents. Pharmamaceuticals. 2023;16:694. 10.3390/ph16050694 PMC10223347 37242477

[47] Anderson LL, Low IK, Banister SD, McGregor IS, Arnold JC. Pharmacokinetics of phytocannabinoid acids and anticonvulsant effect of cannabidiolic acid in a mouse model of Dravet syndrome. J Nat Prod. 2019;82:3047–55. 10.1021/acs.jnatprod.9b00600 31686510

[48] Yinka OS, Olubunmi OP, Zabdiel AA, Oladele OJ, Taiye AS, Ayodele A, et al. Peroral exposure to Cannabis sativa a ethanol extract caused neuronal degeneration and astrogliosis in wistar rats’ prefrontal cortex. Ann Neurosci. 2023;30:84–95. 10.1177/09727531221120988 PMC10496793 37706104

[49] Haghparast E, Sheibani V, Komeili G, Chahkandi M, Rad NS. The effects of chronic marijuana administration on 6-OHDA-induced learning & memory impairment and hippocampal dopamine and cannabinoid receptors interaction in male rats. Neurochem Res. 2023;48:2220–9. 10.1007/s11064-023-03899-8 36894794

[50] Reisdorph N, Doenges K, Levens C, Manke J, Armstrong M, Smith H, et al. Oral cannabis consumption and intraperitoneal THC: CBD dosing results in changes in brain and plasma neurochemicals and endocannabinoids in mice. J Cannabis Res. 2024;6:10. 10.1186/s42238-024-00219-x PMC10908076 38429800

[51] Burggren AC, Shirazi A, Ginder N, London ED. Cannabis effects on brain structure, function, and cognition: considerations for medical uses of cannabis and its derivatives. Am J Drug Alcohol Abuse. 2019;45:563–79. 10.1080/00952990.2019.1634086 PMC7027431 31365275

[52] ElShebiney S, El-Denshary ES, Abdel-Salam O, Salem NA, El-Khyat ZA, El N. Cannabis resin extract in Parkinson’s disease: behavioral, neurochemical, and histological evaluation. Cell Biol Res Ther. 2014;10:12.

[53] Dosumu OA, Taiwo OA, Akinloye OA, Obadina AO, Rotimi SO, Owolabi OP, et al. Implications of Cannabis sativa on serotonin receptors 1B (HTR1B) and 7 (HTR7) genes in modulation of aggression and depression. Vegetos. 2022;35:19–25.

[54] Wang M, Wang Y-H, Avula B, Radwan MM, Wanas AS, van Antwerp J, et al. Decarboxylation study of acidic cannabinoids: a novel approach using ultra-high-performance supercritical fluid chromatography/photodiode array-mass spectrometry. Cannabis Cannabinoid Res. 2016;1:262–71. 10.1089/can.2016.0020 PMC5549281 28861498

[55] Abdel-Salam OM, El-Shamarka ME-S, Salem NA, Gaafar AE-DM. Effects of Cannabis sativa extract on haloperidol-induced catalepsy and oxidative stress in the mice. Excli J. 2012;11:45. PMC4928014 27366134

[56] Amaza DS, Maidugu F, Zirahei J, Numan A, Mari H. The effect of Cannabis sativa leaves aqueous extract on cerebral cortex in albino rats. J Dental Med Sci. 2013;6:53–58.

[57] Schwarz AM, Keresztes A, Bui T, Hecksel RJ, Peña A, Lent B, et al. Terpenes from Cannabis sativa induce antinociception in mouse chronic neuropathic pain via activation of spinal cord adenosine A(2A) receptors. bioRxiv. 2023. 10.1097/j.pain.0000000000003265 PMC11511650 38709489

[58] Demshimeno P, Ukoha U, Aguwa U. Effect of aqueous extract of Cannabis sativa leaf on the oxidative stress markers in the brain of male wistar rats. Asian J Appl Chem Res. 2024;15:53–62.

[59] Riedel G, Fadda P, McKillop‐Smith S, Pertwee RG, Platt B, Robinson L. Synthetic and plant‐derived cannabinoid receptor antagonists show hypophagic properties in fasted and non‐fasted mice. Br J Pharmacol. 2009;156:1154–66. 10.1111/j.1476-5381.2008.00107.x PMC2697695 19378378

[60] Abey NO. Cannabis sativa (Marijuana) alters blood chemistry and the cytoarchitecture of some organs in Sprague Dawley rat models. Food Chem Toxicol. 2018;116:292–97. 10.1016/j.fct.2018.04.023 29679609

[61] Weinstein A, Brickner O, Lerman H, Greemland M, Bloch M, Lester H, et al. A study investigating the acute dose–response effects of 13 mg and 17 mg Δ 9-tetrahydrocannabinol on cognitive–motor skills, subjective and autonomic measures in regular users of marijuana. J Psychopharmacol. 2008;22:441–51. 10.1177/0269881108088194 18635724

[62] Ahmed Z, Isaq H, Khan ZS, Noureen H, Khan MA, Ishaq M. Effect of Cannabis sativa extract on the liver and cardic enzymes of normal healthy mice. Khyber Med Univ J. 2016;9:117.

[63] Mobashar A, Akbar Z, Barkat K, Hussain K, Nadeem H, Kharl HAA, et al. Evaluation of anti-inflammatory and anti-arthritic activities of benzimidazole derivative 2-((1H-benzo [d] imiadazol-2-yl) thio)-1-3, 5-diphenyl-1h-pyrazol-1-yl) ethanone. Atl J Life Sci. 2025;2025.

[64] Kumar R, Chambers W, Pertwee R. Pharmacological actions and therapeutic uses of cannabis and cannabinoids. Anaesthesia. 2001;56:1059–68. 10.1046/j.1365-2044.2001.02269.x 11703238

[65] Scherberich JE, Gruber R, Nockher WA, Christensen EI, Schmitt H, Herbst V, et al. Serum uromodulin—a marker of kidney function and renal parenchymal integrity. Nephrol Dialysis Transplant. 2018;33:284–95. 10.1093/ndt/gfw422 PMC5837243 28206617

[66] Filipiuc L-E, Ştefănescu R, Solcan C, Ciorpac M, Szilagyi A, Cojocaru D, et al. Acute toxicity and pharmacokinetic profile of an EU-GMP-certified Cannabis sativa L. in rodents. Pharmaceuticals. 2023;16:694. 10.3390/ph16050694 PMC10223347 37242477

[67] de Faria Viana E, de Carvalho Mello HH, Carvalho FB, Café MB, Leandro NSM, Arnhold E, et al. Blood biochemical parameters and organ development of brown layers fed reduced dietary protein levels in two rearing systems. Anim Biosci. 2022;35:444. 10.5713/ab.21.0145 PMC8902232 34293840

[68] Groce E. The health effects of cannabis and cannabinoids: the current state of evidence and recommendations for research; 2018. 28182367

[69] Hill KP, Palastro MD, Johnson B, Ditre JW. Cannabis and pain: a clinical review. Cannabis Cannabinoid Res. 2017;2:96–104. 10.1089/can.2017.0017 PMC5549367 28861509

[70] Deng J, Han J, Chen J, Zhang Y, Huang Q, Wang Y, et al. Comparison of analgesic activities of aconitine in different mice pain models. PLoS One. 2021;16:e0249276. 10.1371/journal.pone.0249276 PMC8016268 33793632

[71] Doncheva ND, Vasileva L, Saracheva K, Dimitrova D, Getova D. Study of antinociceptive effect of ketamine in acute and neuropathic pain models in rats. Adv Clin Exp Med. 2019;28:573–79. 10.17219/acem/94143 30561175

[72] Rapacz A, Rybka S, Obniska J, Jodłowska A, Góra M, Koczurkiewicz P, et al. Analgesic and antiallodynic activity of novel anticonvulsant agents derived from 3-benzhydryl-pyrrolidine-2, 5-dione in mouse models of nociceptive and neuropathic pain. Eur J Pharmacol. 2020;869:172890. 10.1016/j.ejphar.2019.172890 31874144

[73] Sofia RD, Vassar HB, Knobloch LC. Comparative analgesic activity of various naturally occurring cannabinoids in mice and rats. J Psychopharmacol. 1975;40:285–95. 10.1007/BF00421466 1170585

[74] Mlost J, Bryk M, Starowicz K. Cannabidiol for pain treatment: focus on pharmacology and mechanism of action. Int J Mol Sci. 2020;21:8870. 10.3390/ijms21228870 PMC7700528 33238607

[75] Abraham AD, Leung EJ, Wong BA, Rivera ZM, Kruse LC, Clark JJ, et al. Orally consumed cannabinoids provide long-lasting relief of allodynia in a mouse model of chronic neuropathic pain. J Neuropsychopharmacol. 2020;45:1105–14. 10.1038/s41386-019-0585-3 PMC7235274 31812152

[76] Tham M, Yilmaz O, Alaverdashvili M, Kelly ME, Denovan‐Wright EM, Laprairie RB. Allosteric and orthosteric pharmacology of cannabidiol and cannabidiol‐dimethylheptyl at the type 1 and type 2 cannabinoid receptors. Br J Pharmacol. 2019;176:1455–69. 10.1111/bph.14440 PMC6487556 29981240

[77] Martínez-Pinilla E, Varani K, Reyes-Resina I, Angelats E, Vincenzi F, Ferreiro-Vera C, et al. Binding and signaling studies disclose a potential allosteric site for cannabidiol in cannabinoid CB2 receptors. Front Pharmacol. 2017;8:744. 10.3389/fphar.2017.00744 PMC5660261 29109685

[78] Cascio MG, Gauson LA, Stevenson LA, Ross RA, Pertwee RG. Evidence that the plant cannabinoid cannabigerol is a highly potent α2‐adrenoceptor agonist and moderately potent 5HT1A receptor antagonist. Br J Pharmacol. 2010;159:129–41. 10.1111/j.1476-5381.2009.00515.x PMC2823359 20002104

[79] Nachnani R, Raup-Konsavage WM, Vrana KE. The pharmacological case for cannabigerol. J Pharmacol Exp Ther. 2021;376:204–12. 10.1124/jpet.120.000340 33168643

[80] Bolognini D, Costa B, Maione S, Comelli F, Marini P, Di Marzo V, et al. The plant cannabinoid Δ9‐tetrahydrocannabivarin can decrease signs of inflammation and inflammatory pain in mice. Br J Pharmacol. 2010;160:677–87. 10.1111/j.1476-5381.2010.00756.x PMC2931567 20590571

[81] Kalvala AK, Bagde A, Arthur P, Surapaneni SK, Ramesh N, Nathani A, et al. Role of cannabidiol and tetrahydrocannabivarin on paclitaxel-induced neuropathic pain in rodents. Int Immunopharmacol. 2022;107:108693. 10.1016/j.intimp.2022.108693 PMC10791145 35303507

[82] Aguilar-Avila DS, Flores-Soto ME, Tapia-Vázquez C, Pastor-Zarandona OA, López-Roa RI, Viveros-Paredes JM. β-Caryophyllene, a natural sesquiterpene, attenuates neuropathic pain and depressive-like behavior in experimental diabetic mice. J Med Food. 2019;22:460–8. 10.1089/jmf.2018.0157 30864870

[83] Aly E, Khajah MA, Masocha W. β-Caryophyllene, a CB2-receptor-selective phytocannabinoid, suppresses mechanical allodynia in a mouse model of antiretroviral-induced neuropathic pain. Molecules. 2019;25:106. 10.3390/molecules25010106 PMC6983198 31892132

[84] Eeswara A, Pacheco-Spiewak A, Jergova S, Sagen J. Combined non-psychoactive Cannabis components cannabidiol and β-caryophyllene reduce chronic pain via CB1 interaction in a rat spinal cord injury model. PLoS One. 2023;18:e0282920. 10.1371/journal.pone.0282920 PMC10010563 36913400

[85] Sofia RD, Barry H. Acute and chronic effects of Δ 9-tetrahydrocannabinol on food intake by rats. Psychopharmacology. 1974;39:213–22. 10.1007/BF00421028 4427988

[86] Boyaji S, Merkow J, Elman RNM, Kaye AD, Yong RJ, Urman RD. The role of cannabidiol (CBD) in chronic pain management: an assessment of current evidence. Curr Pain Headache Rep. 2020;24:1–6. 10.1007/s11916-020-0835-4 31980957

[87] Casey SL, Atwal N, Vaughan CW. Cannabis constituent synergy in a mouse neuropathic pain model. Pain. 2017;158:2452–60. 10.1097/j.pain.0000000000001051 28885457

